# Supplementary material for: Alteration in the Synaptic and Extrasynaptic Organization of AMPA Receptors in the Hippocampus of P301S Tau Transgenic Mice
Source: Int J Mol Sci. 2022 Nov 4;23(21):13527. doi: 10.3390/ijms232113527 (PMC9656470; doi:10.3390/ijms232113527)
Supplement: Supplementary file 1 [file ijms-23-13527-s001.zip › ijms-1979043-SI.pdf]

Supplementary Information

Alteration in the synaptic and extrasynaptic organization of AMPA receptors in the hippocampus of P301S tau transgenic mice

Rocio Alfaro-Ruíz[1], Carolina Aguado[1], Alejandro Martín-Belmonte[1,2,3], Ana Esther Moreno-Martínez[1], Jesús Merchán-Rubira[5], Félix Hernández[4,5], Jesús Ávila[4,5], Yugo Fukazawa[6] and Rafael Luján[1]

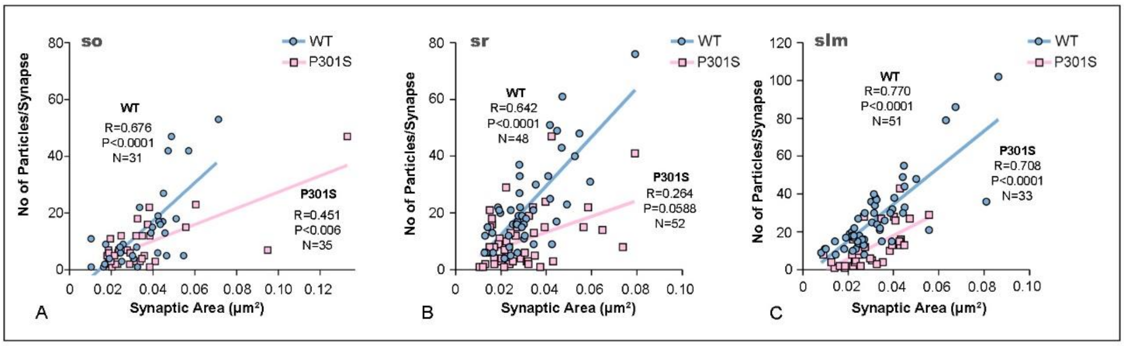

Supplementary Figure S1. ***GluA1-4 immunoparticle density at excitatory synapses on spines***. Correlation of the number of GluA1-4 immunoparticles and IMP-cluster area on pyramidal cell spines. (A-C) Scatter plots showing the correlation between surface areas of postsynaptic membrane specializations and numbers of gold particles labelling GluA1-4 receptors in the three dendritic layers in both wild type and P301S mice. A positive linear correlation between immunoparticle number and synaptic size was detected throughout the CA1 field (Spearman's rank-order correlation).

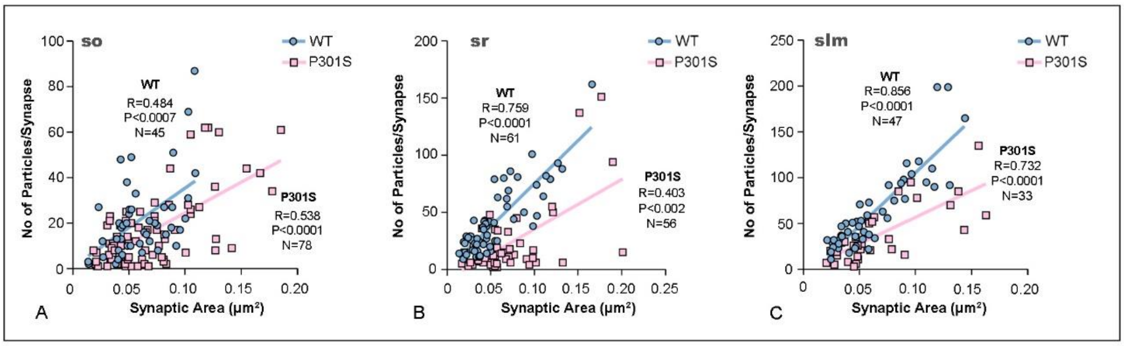

Supplementary Figure S2. ***GluA1-4 immunoparticle density at excitatory synapses on interneurons***. Correlation of the number of GluA1-4 immunoparticles and IMP-cluster area on interneuron dendrites. (A-C) Scatter plots showing the correlation between surface areas of postsynaptic membrane specializations and numbers of gold particles labelling GluA1-4 receptors in the three dendritic layers in both wild type and P301S mice. In the three dendritic layers there is a positive linear correlation between immunoparticle number and synaptic size (Spearman's rank-order correlation).
